# Natural aminosterols inhibit NMDA receptors with low nanomolar potency

**DOI:** 10.1111/febs.70072

**Published:** 2025-03-24

**Authors:** Giulia Fani, Elisabetta Coppi, Silvia Errico, Federica Cherchi, Martina Gennari, Denise Barbut, Michele Vendruscolo, Michael Zasloff, Anna Maria Pugliese, Fabrizio Chiti

**Affiliations:** ^1^ Department of Experimental and Clinical Biomedical Sciences, Section of Biochemistry University of Florence Italy; ^2^ Department of Neuroscience, Psychology, Drug Research and Child Health (NEUROFARBA), Division of Pharmacology and Toxicology University of Florence Italy; ^3^ Centre for Misfolding Diseases, Yusuf Hamied Department of Chemistry University of Cambridge UK; ^4^ Enterin Research Institute Inc. Philadelphia PA USA; ^5^ MedStar‐Georgetown Transplant Institute Georgetown University School of Medicine Washington DC USA

**Keywords:** ENT‐03, ionotropic glutamate receptors, negative allosteric modulators, NMDA antagonist, squalamine

## Abstract

Abnormal functions of N‐methyl‐D‐aspartate receptors (NMDARs) are associated with many brain disorders, making them primary targets for drug discovery. We show that natural aminosterols inhibit the NMDAR‐mediated increase of intracellular calcium ions in cultured primary neurons and neuroblastoma cells. Structural comparison with known NMDAR‐negative allosteric modulators, such as pregnanolone‐sulfate‐2 (PAS), raises the hypothesis that aminosterols have the same mechanism of action. Fluorescence resonance energy transfer (FRET) measurements using labeled NMDAR and the labeled aminosterol trodusquemine (TRO) indicate close spatial proximity, likely arising from binding. Other indirect yet plausible mechanisms for NMDAR inhibition by TRO were excluded. Electrophysiological patch clamp measurements on primary neurons indicate that pre‐incubated TRO inhibits NMDA‐induced ion currents with a *IC*_50_ of 5 nm. Inhibition is observed only after cell membrane pre‐adsorption, indicating accessibility to NMDAR from the cell membrane and binding to the transmembrane domains (TMDs) and TMD‐ligand‐binding domain (LBD) linkers, similarly to PAS. The TRO IC_50_ is 5000‐fold higher than that of PAS and 20–16 000 times higher than those of other inhibitors binding to TMD/TMD‐LBD regions, identifying aminosterols as promising and potent NMDAR modulators.

## Graphical abstract

Misfunction of N‐methyl‐D‐aspartate (NMDA) receptors is associated with many brain disorders, making them primary targets for drug discovery. Trodusquemine and other natural aminosterols inhibit NMDA receptors with low nanomolar potency through binding to the cell membranes followed by interaction with the channels. The IC_50_ value is 5000‐fold higher than that of the structurally related pregnanolone‐sulfate‐2 (PAS) and 20–16 000 times higher than inhibitors binding to the TMD/TMD‐LBD regions of the receptors.

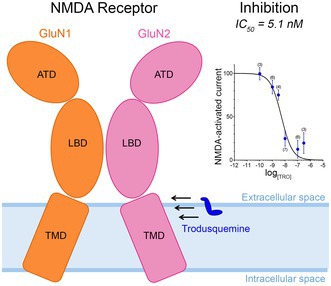

**Abbreviations**
AA: arachidonic acid
AD: Alzheimer's disease
ALS: amyotrophic lateral sclerosis
AMPARs: α‐amino‐3‐hydroxy‐5‐methyl‐4‐isoxazolepropionic acid receptors
ARA‐C: cytosine β‐d‐arabinofuranoside hydrochloride
ATD: amino‐terminal domain
Aβ: amyloid β
BD: bipolar disorder
Ca2+: calcium ions
CHOL: cholesterol
Cm: membrane capacitance
CM‐H2DCFDA: 2′,7′‐dichlorodihydrofluorescein diacetate
CNS: central nervous system
CTD: carboxyl‐terminal domain
DMEM: Dulbecco's modified Eagle's medium
FBS: fetal bovine serum
FRET: Fluorescence resonance energy transfer
GM1: monosialotetrahexosylganglioside 1
HD: Huntington's disease
HEPES: N‐2‐hydroxyethylpiperazine‐N‐2‐ethanesulfonic acid
IC50: half maximal inhibitory concentration
IS: ischemic stroke
KARs: kainate receptors
LBD: ligand‐binding domain
LPC: lysophosphatidylcholine
LPS: lipopolysaccharides
LTD: long‐term depression
LUVs: large unilamellar vesicles
MDD: major depressive disorder
Mem: memantine
NGF: nerve growth factor
NMDARs: N‐methyl‐d‐aspartate receptors
NP: neuropathic pain
PAS: pregnanolone‐sulfate‐2
PD: Parkinson's disease
PTP1B: protein‐tyrosine phosphatase 1B
ROS: reactive oxygen species
Rs: series resistance
SAR: structure–activity relationship
SC: schizophrenia
SEM: standard error of the mean
SQ: squalamine
TMD: transmembrane domains
TRO: trodusquemine
TTX: tetrodotoxin
αS: α‐synuclein

## Introduction

N‐methyl‐d‐aspartate receptors (NMDARs) are important ionotropic glutamate receptors present on neuronal cell membranes [1, 2, 3]. They are highly permeable to calcium ions (Ca^2+^) and physiologically activated by glutamate as an agonist and glycine or D‐serine as a co‐agonist, allowing a flux of Ca^2+^ across the cell membrane, from the extracellular space to the cytosol [1, 2, 3]. NMDARs control critical events in excitatory transmission, formation, and development of synaptic organization and synaptic plasticity in the central nervous system (CNS), but their overactivation can promote neuronal death in various neuropathological conditions. It is now recognized that an abnormal Ca^2+^ influx mediated by NMDAR hyperactivation is associated with Alzheimer's disease (AD), Parkinson's disease (PD), Huntington's disease (HD), amyotrophic lateral sclerosis (ALS), schizophrenia (SC), major depressive disorder (MDD), bipolar disorder (BD), neuropathic pain (NP), ischemic stroke (IS), epilepsy, and other brain disorders [4, 5, 6, 7, 8, 9].

NMDARs are membrane‐embedded hetero‐tetramers [1, 2, 3, 10]. To date, seven subunits have been identified, namely GluN1 (containing eight alternatively spliced isoforms), GluN2A‐D, and GluN3A‐B, all sharing four distinct semiautonomous domains: the extracellular amino‐terminal domain (ATD), the extracellular ligand‐binding domain (LBD), the transmembrane domain (TMD) containing three transmembrane segments (M1, M3, and M4), and a re‐entrant pore loop (M2), and an intracellular carboxyl‐terminal domain (CTD). In this structure, the LBD contains two portions between the ATD and M1 (S1) and between M3 and M4 (S2). The M2 loop determines the Ca^2+^ flux and mediates Mg^2+^ blockade. Most native NMDARs contain two glycine‐binding subunits GluN1 and two glutamate‐binding subunits GluN2. Following the association of NMDARs with neurological diseases, a large number of NMDAR inhibitors and potentiators have been reported, many of which are selective for well‐defined subunit types and have binding sites to specific domains or domain interfaces [11, 12, 13].

**Fig. 1:**
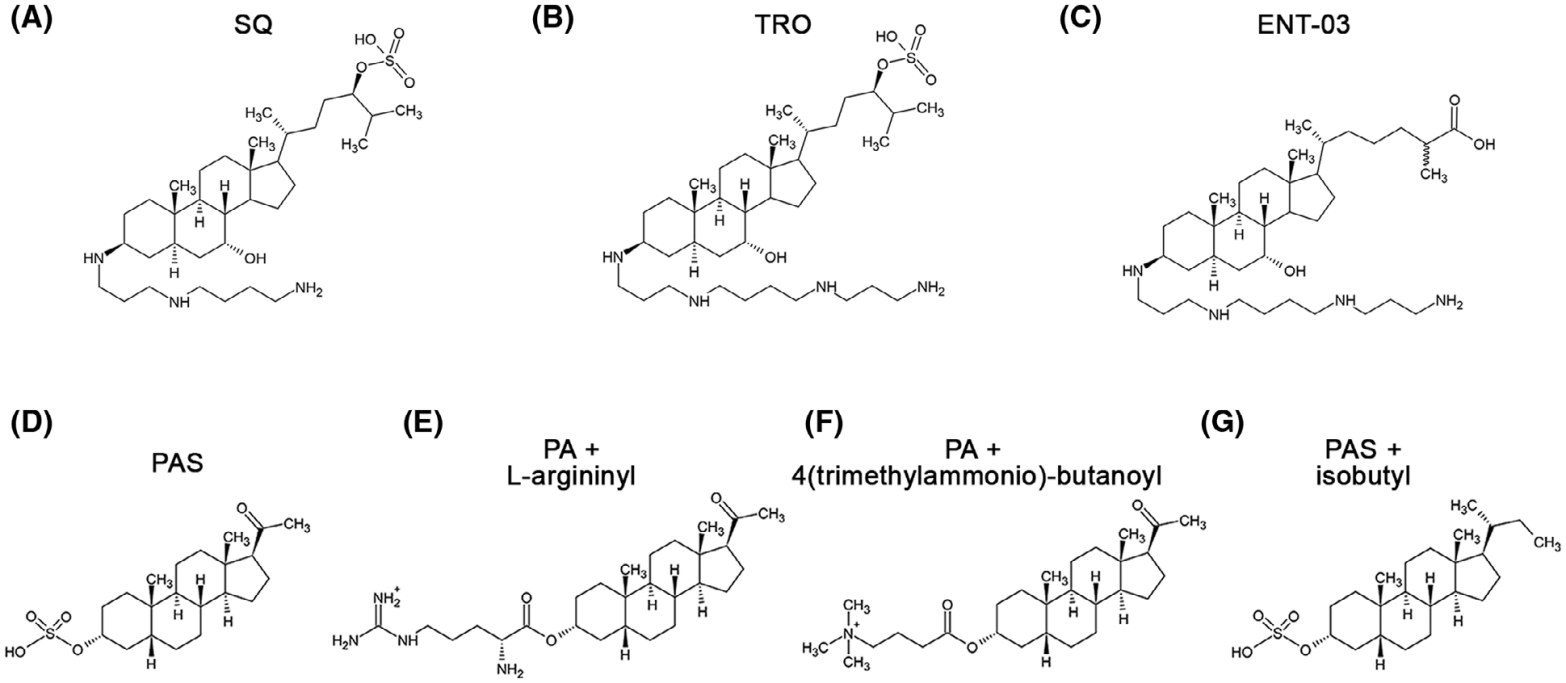
Chemical structures of aminosterols, pregnanolone‐sulfate‐2 (PAS), and derivatives. Chemical structures of squalamine (SQ, A), trodusquemine (TRO, B), ENT‐03 (C), PAS (D), PA with L‐argininyl replacing the sulfate group at C3 (E), PA with 4(trimethylammonio)‐butanoyl replacing the sulfate group at C3 (F), PAS with isobutyl group replacing the acetyl group at C17 (G).

From the observation that the chemical structure of natural aminosterols resembles that of well‐known NMDAR inhibitors (see below), we hypothesized that certain aminosterols may act as putative inhibitors of NMDARs and, therefore, can be investigated as potential therapeutic compounds against the various pathological conditions associated with NMDAR abnormal activation. The three natural aminosterols that have been studied most extensively are squalamine (SQ, also known as ENT‐01), trodusquemine (TRO, also known as ENT‐02 or MSI‐1436), originally isolated from the dogfish shark *Squalus acanthias* [14, 15], and ENT‐03, recently isolated from the mouse *Mus musculus* [16] (Fig. 1A–C). They have in common a steroidal skeleton, an alkyl moiety of the cholestane type fused to the sterol C‐17 of the D‐ring and an alkyl polyamine tail fused to the sterol C‐3 of the A‐ring and replacing the hydroxyl group (Fig. 1A–C), as previously reported [15, 16, 17].

Despite the many beneficial effects observed for aminosterols against neurodegenerative conditions such as PD and AD [18], these compounds have never been tested as NMDAR inhibitors, and no evidence has been reported so far in this direction to our knowledge. Nevertheless, all three aminosterols have a central portion of their structure reminiscent of that of the natural widely studied NMDAR inhibitor pregnanolone‐sulfate‐2, whose complete name is 20‐oxo‐5β‐pregnan‐3α‐yl sulfate, commonly abbreviated as 3α5βS, PAS, or PA‐S (Fig. 1D) [11, 19]. Unlike PAS, aminosterols have a positively charged alkyl polyamine tail fused to C‐3 of the sterol moiety, as opposed to the negatively charged sulfate ester in the corresponding position for PAS, and an alkyl moiety of the cholestane type fused to C‐17 in the D‐ring, as opposed to the acetyl group in the corresponding position for PAS (Fig. 1A–D). Nevertheless, structure–activity relationship (SAR) studies of PAS established that any positively charged group to replace the negatively charged sulfate group at C‐3, including L‐argininyl to form PA‐27 (Fig. 1E) and 4(trimethylammonio)‐butanoyl to form PA‐35 (Fig. 1F), maintain, and even augment, the NMDAR inhibitory activity of PAS [11, 20]. Elongation of this tail with introduction of a progressively higher number of methylene groups, while maintaining the charge, also potentiates inhibition [21, 22]. In addition, replacement of the acetyl group on the other side of the molecule (C‐17) with more hydrophobic alkyl groups, such as an isobutyl group (Fig. 1G), increases the inhibitory activity of PAS remarkably [23].

These arguments on the SAR of PAS suggested that these natural aminosterols may act as high‐affinity inhibitors of NMDARs. Using cell biological, biophysical, and electrophysiological methods, we report that TRO abolishes the NMDA‐induced flux of Ca^2+^ across the cell membrane of neuroblastoma and primary neurons and appears to be a potent ligand inhibitor of NMDARs, with a half maximal inhibitory concentration (IC_50_) value estimated on primary neurons and electrophysiological methods of about 5 nm. These results position TRO as a potent NMDAR inhibitor and as the most potent among those binding to the TMDs and TMD‐LBD linkers so far discovered.

## Results

### TRO inhibits NMDAR activation induced by its agonist NMDA

**Fig. 2:**
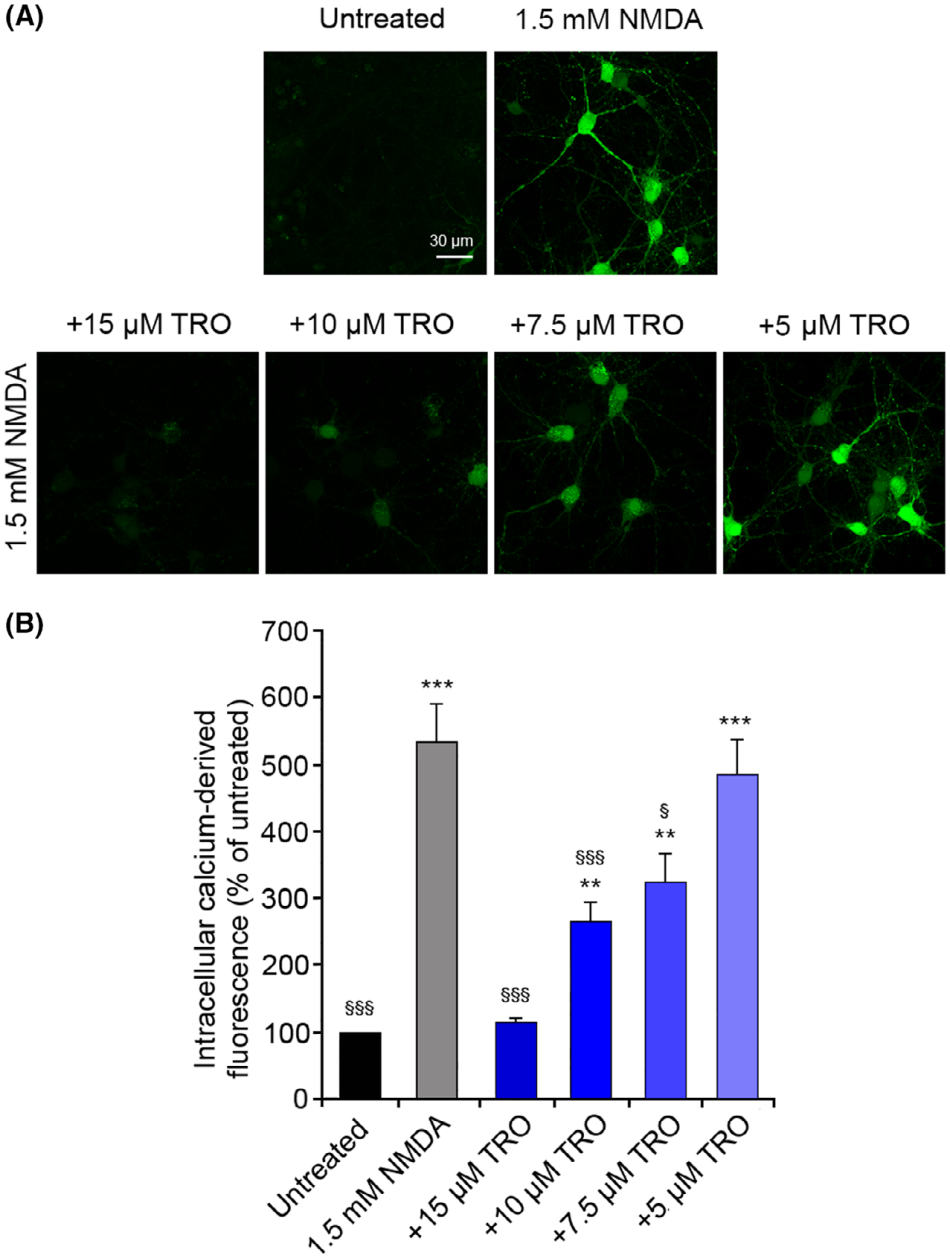
Intracellular free Ca^2+^ levels in primary rat cortical neurons treated with N‐methyl‐d‐aspartate (NMDA) after treatment with different trodusquemine (TRO) concentrations. (A) Representative confocal scanning microscopy images of untreated cells and cells treated for 10 min with 1.5 mm NMDA without or with pretreatment with 15, 10, 7.5, and 5 μm TRO for 10 min. Scale bar: 30 μm. (B) Semi‐quantitative analysis of intracellular free Ca^2+^‐derived fluorescence as shown in panel A. Variable numbers of cells (12–22) in three different experiments (*n* = 3) were analyzed for each condition, error bars are SEM. Comparisons between the different groups were performed by Student's *t*‐test, double (**) and triple (***) asterisks refer to *P* values <0.01 and <0.001, respectively, relative to untreated cells. Single (§) and triple (§§§) symbols refer to *P* values <0.05 and <0.001, respectively, relative to NMDA treatment.

We initially monitored the change of intracellular Ca^2+^ levels in primary rat cortical neurons treated with 1.5 mm NMDA, which is a selective agonist of NMDARs, in the absence and presence of different concentrations of TRO. The results showed a significant increase of intracellular Ca^2+^ levels in cells treated with NMDA, compared to untreated cells (Fig. 2). Such an increase disappeared when the NMDA treatment was preceded by a 10 min pretreatment with 15 μm TRO, with levels of Ca^2+^ similar to the untreated cells. The TRO‐mediated inhibition of Ca^2+^ influx was dose‐dependent, as it was reduced at lower TRO concentrations, until reaching no inhibitory effect with 5 μm TRO (Fig. 2). These results suggest an ability of TRO to abolish the NMDA‐induced flux of Ca^2+^ across the cellular membrane in primary rat cortical neurons.

**Fig. 3:**
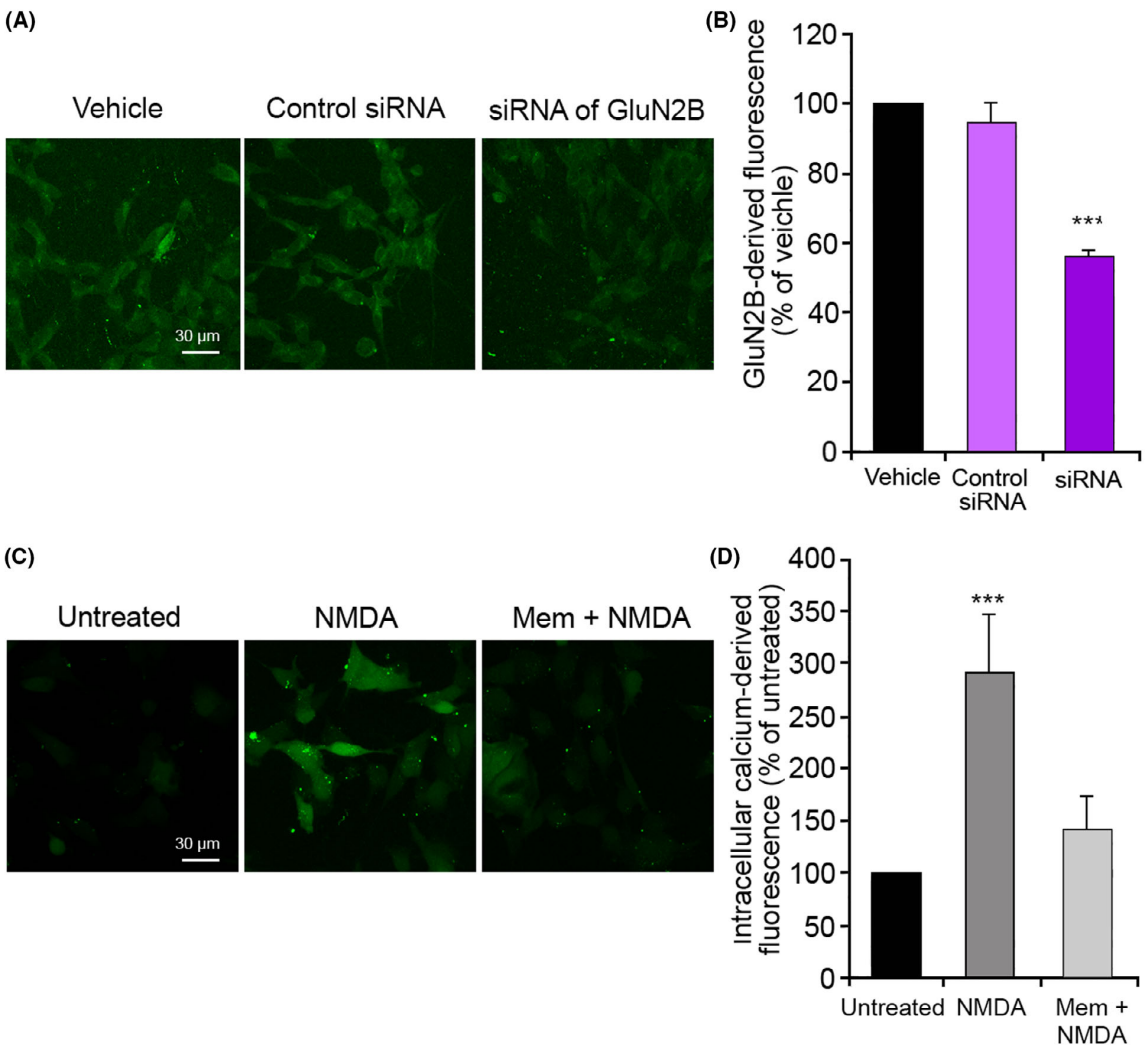
Presence of functionally active N‐methyl‐d‐aspartate receptors (NMDARs) in SH‐SY5Y cells. (A) GluN2B‐derived fluorescence in representative confocal scanning microscopy images of SH‐SY5Y cells treated with a primary antibody against GluN2B, labeled with Alexa Fluor‐488, following pretreatment with vehicle, 25 nm negative control siRNA, and 25 nm siRNA against GluN2B. Scale bar: 30 μm. (B) Semi‐quantitative analysis of GluN2B‐derived fluorescence as shown in panel A. (C) Representative confocal scanning microscopy images of intracellular Ca^2+^ levels in untreated cells and cells treated with 1.5 mm NMDA for 10 min without and with pretreatment with 10 μm memantine (Mem). Scale bar: 30 μm. (D) Semi‐quantitative analysis of intracellular free Ca^2+^‐derived fluorescence as shown in panel C. Variable numbers of cells (12–22) in three different experiments (*n* = 3) were analyzed for each condition, error bars are SEM. Comparisons between the different groups were performed by Student's *t*‐test, triple (***) asterisks refer to *P* values <0.001, relative to vehicle or untreated cells.

**Fig. 4:**
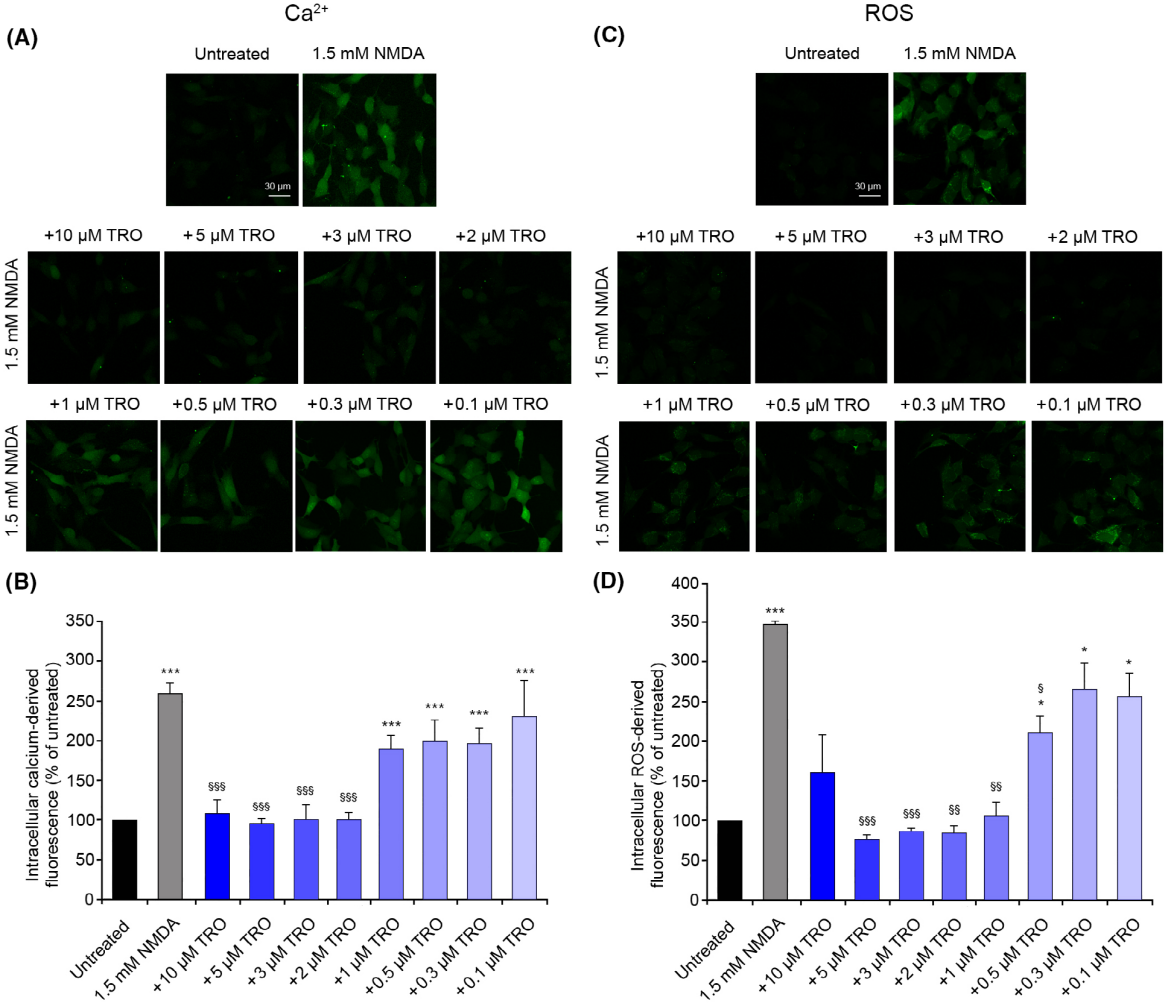
Intracellular free Ca^2+^ levels and ROS levels in SH‐SY5Y cells treated with N‐methyl‐d‐aspartate (NMDA) after treatment with different trodusquemine (TRO) concentrations. (A, C) Representative confocal scanning microscopy images of (A) intracellular Ca^2+^ levels and (C) intracellular ROS levels in untreated cells and cells treated with 1.5 mm NMDA for 10 min (A) and 30 min (B), without or with pretreatment with 10, 5, 3, 2, 1, 0.5, 0.3, and 0.1 μm TRO for 10 min. Scale bar: 30 μm. (B, D) Semi‐quantitative analysis of (B) intracellular free Ca^2+^‐derived fluorescence and (D) intracellular ROS levels, as shown in panels A and C, respectively. Variable numbers of cells (12–22) in three different experiments (*n* = 3) were analyzed for each condition, error bars are SEM. Comparisons between the different groups were performed by Student's *t*‐test, single (*), double (**), and triple (***) asterisks refer to *P* values <0.05, <0.01, and <0.001, respectively, relative to untreated cells. Single (§), double (§§), and triple (§§§) symbols refer to *P* values <0.05, <0.01, and <0.001, respectively, relative to NMDA treatment.

To investigate the mechanism by which this inhibitory effect occurs, we repeated and expanded our analysis in human SH‐SY5Y neuroblastoma cells, which is a relevant cell model for neurotoxicity studies and can be cultured more easily. Firstly, we checked the expression of NMDARs in this cell line, using a fluorescently labeled primary antibody against the subunit 2B of the NMDAR (GluN2B) and pretreating SH‐SY5Y cells with the siRNA against GluN2B to observe a reduction of the derived fluorescence. A high NMDAR‐derived fluorescence was observed in cells pretreated with vehicle and with a negative control siRNA, unlike cells pretreated with the anti‐GluN2B siRNA where the fluorescence was significantly reduced (Fig. 3A,B), suggesting the presence of NMDARs in this cell line. To confirm the functionality of these receptors, the levels of intracellular Ca^2+^ were evaluated after treatment with NMDA, and we observed a significantly higher level compared to untreated cells, which was significantly reduced with a 1 h pretreatment with memantine, an NMDAR uncompetitive inhibitor (Fig. 3C,D), confirming that NMDARs were present and functional in this cell line. Similarly to observations in primary neurons, the treatment of SH‐SY5Y cells with 1.5 mm NMDA for 10 min induced a significant increase of the intracellular Ca^2+^ levels, compared to the untreated cells (Fig. 4A,B), and the pretreatment for 10 min with TRO caused a dose‐dependent reduction of this influx of Ca^2+^, whose levels were similar to the untreated cells from 2 μm to 10 μm TRO (Fig. 4A,B).

We then focused on reactive oxygen species (ROS) production in SH‐SY5Y cells, using the same treatment and pretreatment described above for the Ca^2+^ influx analysis. The treatment with 1.5 mm NMDA was performed for 30 min, instead of 10 min, because ROS production is a slightly delayed process compared to Ca^2+^ rise [24]. This treatment caused a significant increase of intracellular ROS, compared with untreated cells, which was inhibited with high concentrations of TRO (Fig. 4C,D). These results suggest that TRO is able to reduce the production of ROS induced by an hyperactivation of NMDARs.

### TRO does not inhibit NMDARs via indirect mechanisms

**Fig. 5:**
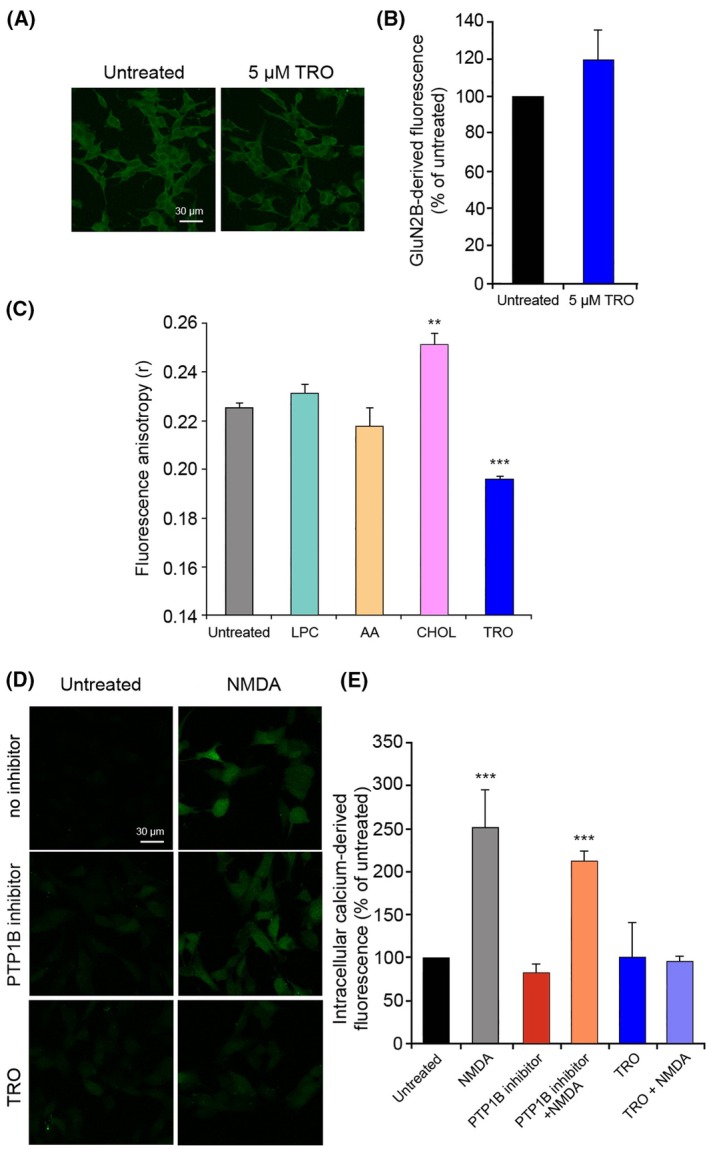
GluN2B expression, TMA‐DPH fluorescence anisotropy, and N‐methyl‐d‐aspartate‐ (NMDA‐) induced intracellular free Ca^2+^ levels in SH‐SY5Y cells treated with trodusquemine (TRO) and other compounds. (A) Representative confocal scanning microscopy images of SH‐SY5Y cells treated with a primary antibody against GluN2B, labeled with Alexa Fluor‐488, in the presence or absence of pretreatment with 5 μm TRO for 10 min. Scale bar: 30 μm. (B) Semi‐quantitative analysis of GluN2B‐derived fluorescence as shown in panel A. (C) Fluorescence anisotropy (*r*) of TMA‐DPH incorporated in the phospholipid bilayer of SH‐SY5Y cell membranes left untreated or treated with 2 μm lysophosphatidylcholine (LPC) for 2 h, 10 μm arachidonic acid (AA) for 2 h, 0.5 mM  cholesterol (CHOL) for 3 h, or 5 μm TRO for 10 min. (D) Representative confocal scanning microscopy images of intracellular Ca^2+^ levels following no treatment (left), or treatment with 1.5 mm NMDA for 10 min (right), with or without pretreatment with 5 μm PTP1B inhibitor for 2 h or 5 μm TRO for 10 min. Scale bar: 30 μm. (E) Semi‐quantitative analysis of intracellular free Ca^2+^‐derived fluorescence as shown in panel D. Variable numbers of cells (12–22) in three different experiments (*n* = 3) were analyzed for each condition, error bars are SEM. Comparisons between the different groups were performed by Student's *t*‐test, double (**) and triple (***) asterisks refer to *P* values <0.01 and <0.001, respectively, relative to untreated cells.

The ability of TRO to inhibit the NMDAR‐mediated Ca^2+^ influx may arise from direct binding of the small molecule with the ionotropic glutamate receptor channel, but may also be due to other indirect mechanisms of action. For example, the removal of NMDARs from the plasma membrane by endocytosis is known to be a mechanism of regulation of the receptor activity, in particular during synapse maturation, during paradigms of long‐term depression (LTD), and in response to ligand binding [25, 26, 27]. To investigate whether NMDAR internalization could represent the mechanism of TRO‐dependent NMDAR inhibition, we probed the receptors on SH‐SY5Y cell membranes with their specific antibodies against the GluN2B subunit, observing their expression on the cell membrane without or with pretreatment with 5 μm TRO for 10 min. Levels of NMDARs did not change following a pretreatment with the aminosterol (Fig. 5A,B), suggesting that this is not the mechanism occurring for its inhibition mediated by TRO.

NMDARs are known to be mechanosensitive, potentiated by mechanical stimuli such as membrane depression, hypotonic solutions and lateral membrane stretch, and inhibited by the opposite stimuli [28, 29]. Changes of the physical properties of the cell membrane, adding specific compounds known to cause its stretching or compression, caused activation or inactivation of NMDARs, respectively [30, 31]. This may well be the case because TRO is well known to bind to the cell membrane [32, 33]. To assess the possibility that TRO inhibits NMDARs through a cell membrane compression mechanism, we analyzed the effects of the compound on the rotational diffusion of a membrane‐embedded TMA‐DPH probe, which is known to insert in the polar head group region of the cell membrane due to its charged group [34]. As controls, we used three different lipids with a known effect on the membrane: lysophosphatidylcholine (LPC) causing a compression of the phospholipid bilayer and neutralizing NMDAR activation, arachidonic acid (AA) exerting an opposite effect following a lipid bilayer stretch with spontaneous NMDAR activation [28, 30, 35, 36, 37, 38], and cholesterol (CHOL) stiffening the membrane [39] and therefore used as a positive control of rotational diffusion reduction. As expected, treatment with LPC and AA caused a slight increase and reduction of the TMA‐DPH anisotropy, respectively, compared with untreated cells (Fig. 5C), confirming the compression and stretching mechanism performed by the two lipids, respectively. Furthermore, the ability of CHOL to increase the membrane rigidity was confirmed with a significant increase of the TMA‐DPH anisotropy, indicating a low rotational freedom of the fluorophore (Fig. 5C). If the inhibitory action of TRO on NMDARs was due to a TRO‐mediated compression of the lipid bilayer, one would expect an increase in TMA‐DPH anisotropy, which is not the case (Fig. 5C), therefore excluding this mechanism as a possible explanation for NMDAR inhibition.

TRO is known to inhibit the nonreceptor protein‐tyrosine phosphatase 1B (PTP1B) by a noncompetitive allosteric mechanism [40, 41]. It was recently reported that PTP1B suppresses the activity of presynaptic NMDARs in the hippocampus of hAPP‐J20 mice, an AD model that overexpresses the amyloid precursor protein [42]. Chronic treatment of animals with TRO or genetic ablation of PTP1B in glutamatergic neurons restored normal electrophysiological activity of the hippocampal NMDARs, as well as cognitive behavior and learning. Improvement in this setting was associated with the preservation of specific phosphorylated tyrosine residues on the cytoplasmic C terminus of the GluN2B subunit [42]. In this *in vivo* setting, TRO enhances presynaptic NMDAR activity through an indirect mechanism, namely, inhibition of PTP1B. Hence, to determine whether the inhibitory activity of TRO against PTP1B was responsible for the acute inhibition of NMDAR activity observed in extrasynaptic NMDARs in our cell model, SH‐SY5Y cells were treated with a PTP1B inhibitor (539749, Merck) distinct and chemically very different from TRO. SH‐SY5Y cells pretreated with the alternative PTP1B inhibitor before NMDA showed high levels of Ca^2+^, similar to those observed with NMDA treatment alone (Fig. 5D,E). By contrast, using the same concentration of TRO before the treatment with NMDA, we observed again a significant reduction of Ca^2+^ influx (Fig. 5D,E), suggesting that the TRO‐mediated inhibition of PTP1B is not involved in the inhibitory mechanism of TRO on NMDARs.

### TRO directly interacts with NMDAR

**Fig. 6:**
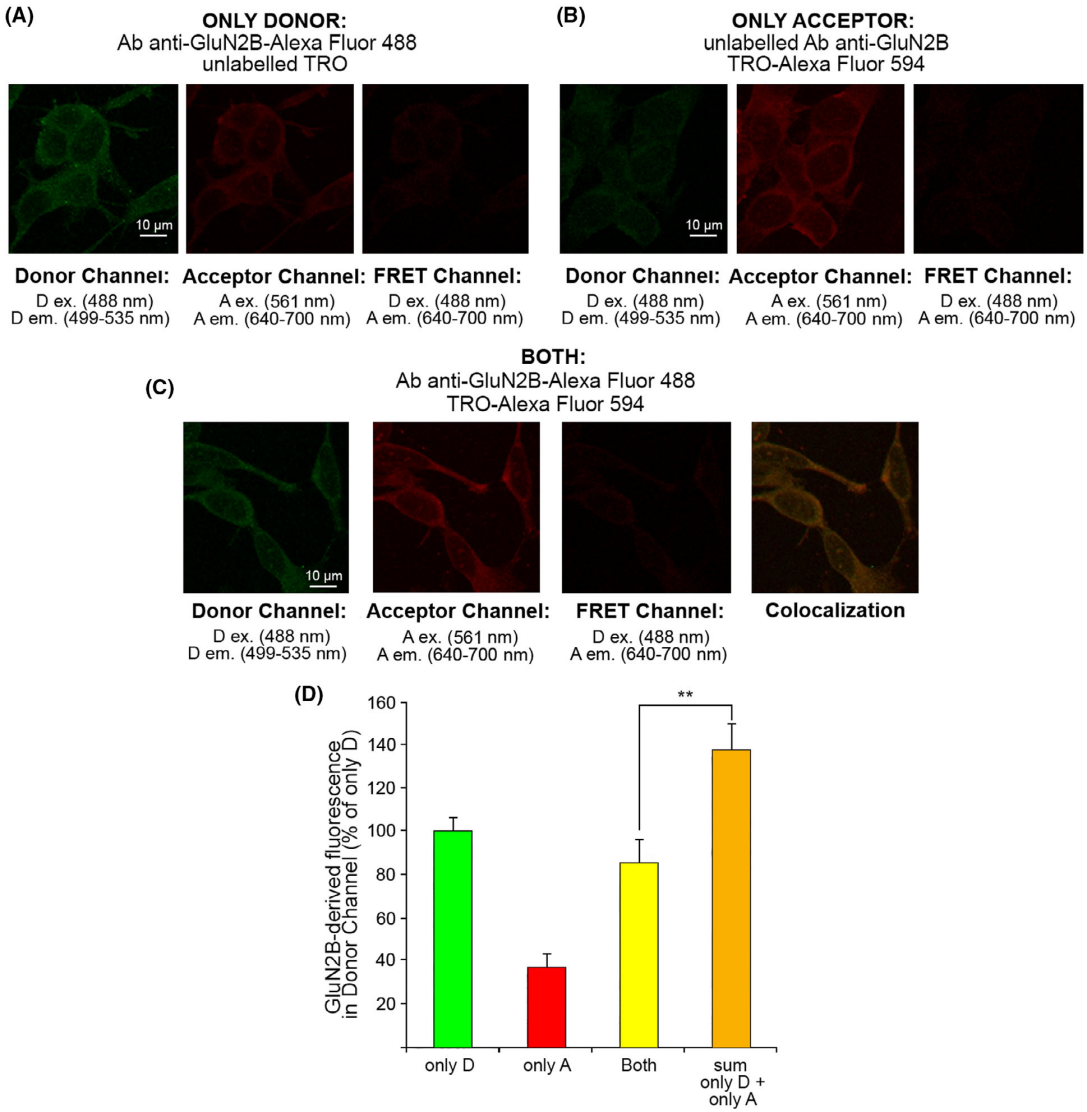
Fluorescence resonance energy transfer (FRET) analysis of the interaction between N‐methyl‐d‐aspartate receptor (NMDAR) and trodusquemine (TRO). (A) Representative confocal scanning microscopy images of SH‐SY5Y cells treated with unlabeled TRO in the presence of anti‐GluN2B antibody labeled with Alexa Fluor‐488 as a donor (D) on the donor channel (left), acceptor channel (middle), and FRET channel (right). Scale bar: 10 μm. (B) Cells treated with TRO labeled with Alexa Fluor‐594 as an acceptor (A) in the presence of unlabeled anti‐GluN2B antibody, on the donor channel (left) acceptor channel (middle) and FRET channel (right). Scale bar: 10 μm. (C) Cells treated with both D and A. From left to right: donor channel, acceptor channel, FRET channel, and colocalization image obtained by overlapping the donor and acceptor channels. Scale bar: 10 μm. (D) Semi‐quantitative analysis of the donor channel in the presence of only D (primary antibody against GluN2B labeled with D), only A (TRO labeled with A), both D and A (primary antibody against GluN2B labeled with D and TRO labeled with A), and the mathematical sum of only D and only A values. Variable numbers of cells (12–22) in three different experiments (*n* = 3) were analyzed for each condition. Comparisons between the different groups were performed by Student's *t*‐test, double (**) asterisks refer to *P* values <0.01. Error bars are SEM.

To explore a possible direct interaction between TRO and NMDAR, we used fluorescence resonance energy transfer (FRET). NMDAR was labeled with a primary antibody labeled with Alexa Fluor‐488 as a donor (D), and TRO was labeled with Alexa Fluor‐594 as an acceptor (A). TRO was labeled with A on the distal primary amino group of its polyamine chain, which is known to stick out of cell membranes from previous studies on TRO and does not, therefore, affect the interaction with the cell membranes significantly [32, 33]. In the presence of only D, SH‐SY5Y cells were excited at 488 nm (D ex wavelength) and the D fluorescence was observed in the 499–535 nm range (D em range, Fig. 6A, left). Then, we excited the sample at 561 nm (A ex wavelength) and observed a bland contribution of D emission in the 640–700 nm range (A em range, Fig. 6A, center), which is due to a technical spillover of the D fluorescence that can be detected also at higher wavelengths. By exciting the samples at 488 nm (D ex wavelength), we observed the absence of emission in the 640–700 nm range (A em range, Fig. 6A, right). In the presence of only A, we excited at 488 nm (D ex wavelength) and monitored the emission in the 499–535 nm range (D em range, Fig. 6B, left), where we observed a bland A fluorescence. We then excited at 561 nm (A ex wavelength) and observed the A emission in the 640–700 nm range (A em range, Fig. 6B, center). We finally observed the absence of A emission in the FRET channel in the 640–700 nm range (A em range) exciting at 488 nm (D ex wavelength, Fig. 6B, right). In the presence of both D and A, we analyzed the donor channel (ex 488 nm, em 499–535 nm), the acceptor channel (ex 543 nm, em 560–610 nm), and the FRET channel (ex 488 nm, em 560–610 nm), and finally we overlapped the donor and acceptor channels to obtain the colocalization image (Fig. 6C).

**Fig. 7:**
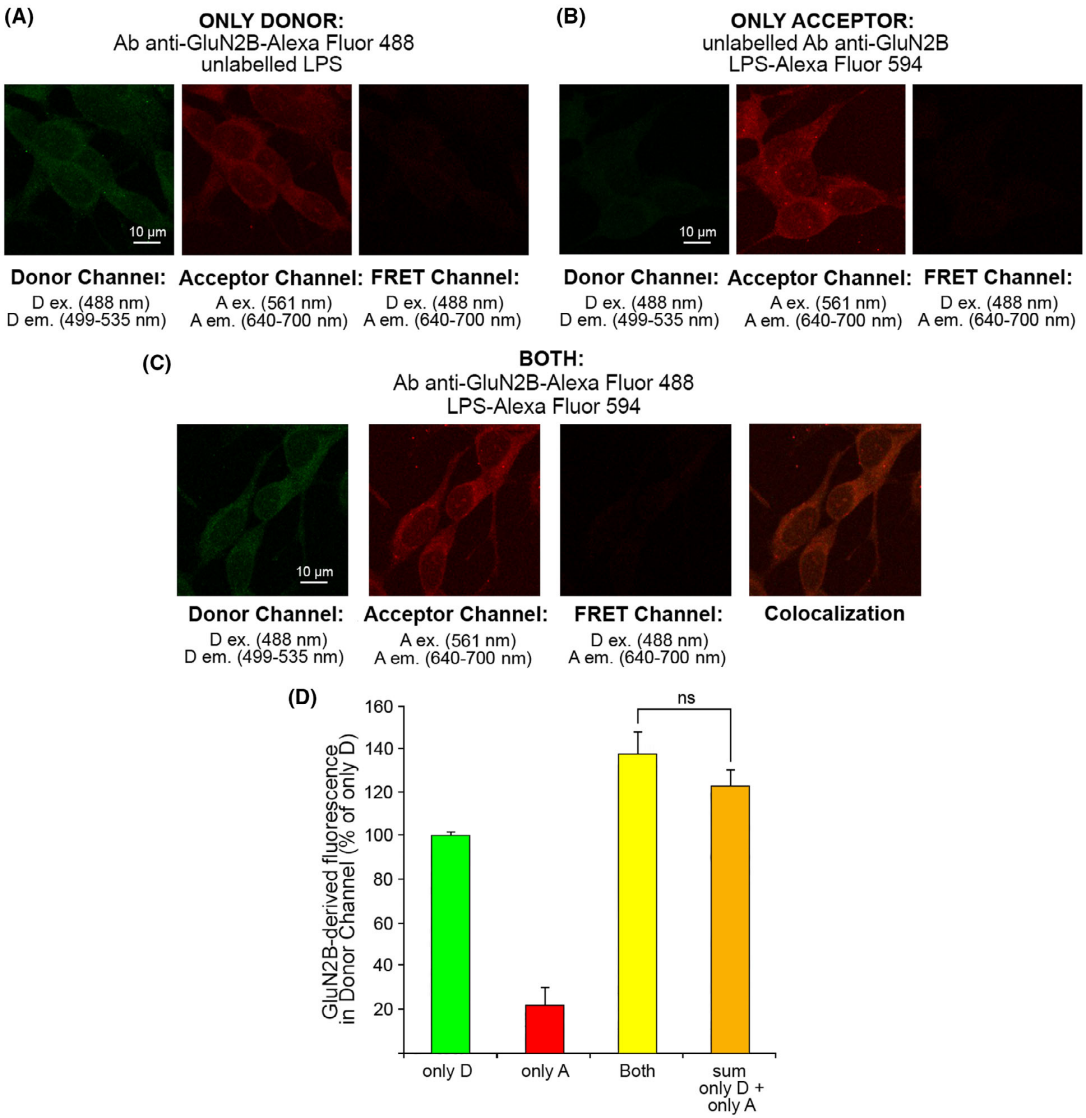
Representative confocal scanning microscopy images of SH‐SY5Y cells for the negative control of fluorescence resonance energy transfer (FRET). (A) Cells treated with unlabeled LPS in the presence of primary antibody against GluN2B labeled with Alexa Fluor‐488 (D) on the donor channel (left, excitation 488 nm, emission 499–535 nm), the acceptor channel (middle, excitation 561 nm, emission 640–700 nm) and the FRET channel (right, excitation 488, emission 640–700 nm). Scale bar: 10 μm. (B) Cells treated with LPS labeled with Alexa Fluor‐594 (A) in the presence of unlabeled primary antibody against GluN2B, on the donor channel (left) the acceptor channel (middle) and the FRET channel (right). Scale bar: 10 μm. (C) Cells treated with both D and A. From left to right, the donor channel, the acceptor channel, the FRET channel, and the colocalization image obtained by overlapping the donor and acceptor channels. Scale bar: 10 μm. (D) Semi‐quantitative analysis of the GluN2B derived fluorescence in the donor channel in the presence of only D (primary antibody against GluN2B labeled with D), only A (LPS labeled with A), both D and A (primary antibody against GluN2B labeled with D and LPS labeled with A), and the sum of the only D fluorescence and only A fluorescence. Variable numbers of cells (12–22) in two different experiments (*n* = 2) were analyzed for each condition. Comparisons between the different groups were performed by Student's *t*‐test, error bars are SEM. n.s., nonsignificant.

We compared the NMDAR‐derived (D‐derived) fluorescence measured in the donor channel in the presence of both D and A with the arithmetic sum of the fluorescence values measured with only D and only A in the donor channel, observing a significant difference (Fig. 6D). By calculating the FRET efficiency (*E*) with Eq. 1 (see Materials and Methods section), we obtained a value of 0.36 ± 0.03, suggesting a direct interaction between TRO and the NMDAR. By contrast, a negative control of FRET using the same anti‐NMDAR antibody labeled with Alexa Fluor‐488 as D and lipopolysaccharides (LPS) labeled with Alexa Fluor‐594 as A, which can be incorporated in the phospholipid bilayer without a direct interaction with NMDARs, did not show any FRET, with an *E* value of −0.1 ± 0.2 (Fig. 7). The FRET analysis, therefore, highlights a direct interaction between TRO and the NMDAR.

### TRO inhibits NMDA‐activated currents with a low nanomolar IC_50_

The results obtained by treating SH‐SY5Y cells with NMDA and various concentrations of TRO and by monitoring the intracellular Ca^2+^ concentrations with a specific probe do not provide an estimate of the TRO *IC*_*50*_ on NMDARs, because Ca^2+^ pumps act by restoring the small intracellular Ca^2+^ concentration. In addition, SH‐SY5Y cells contain small amounts of NMDARs relative to neurons. For this reason, we performed electrophysiological patch clamp experiments on isolated rat hippocampal neurons by measuring directly the effect of different TRO concentrations on NMDAR‐mediated currents activated by NMDA (100 μm) in the presence of its co‐agonist glycine (10 μm). Since we used NMDA rather than glutamate, the agonist‐mediated currents are mainly from NMDARs rather than other ionotropic glutamate receptors.

**Fig. 8:**
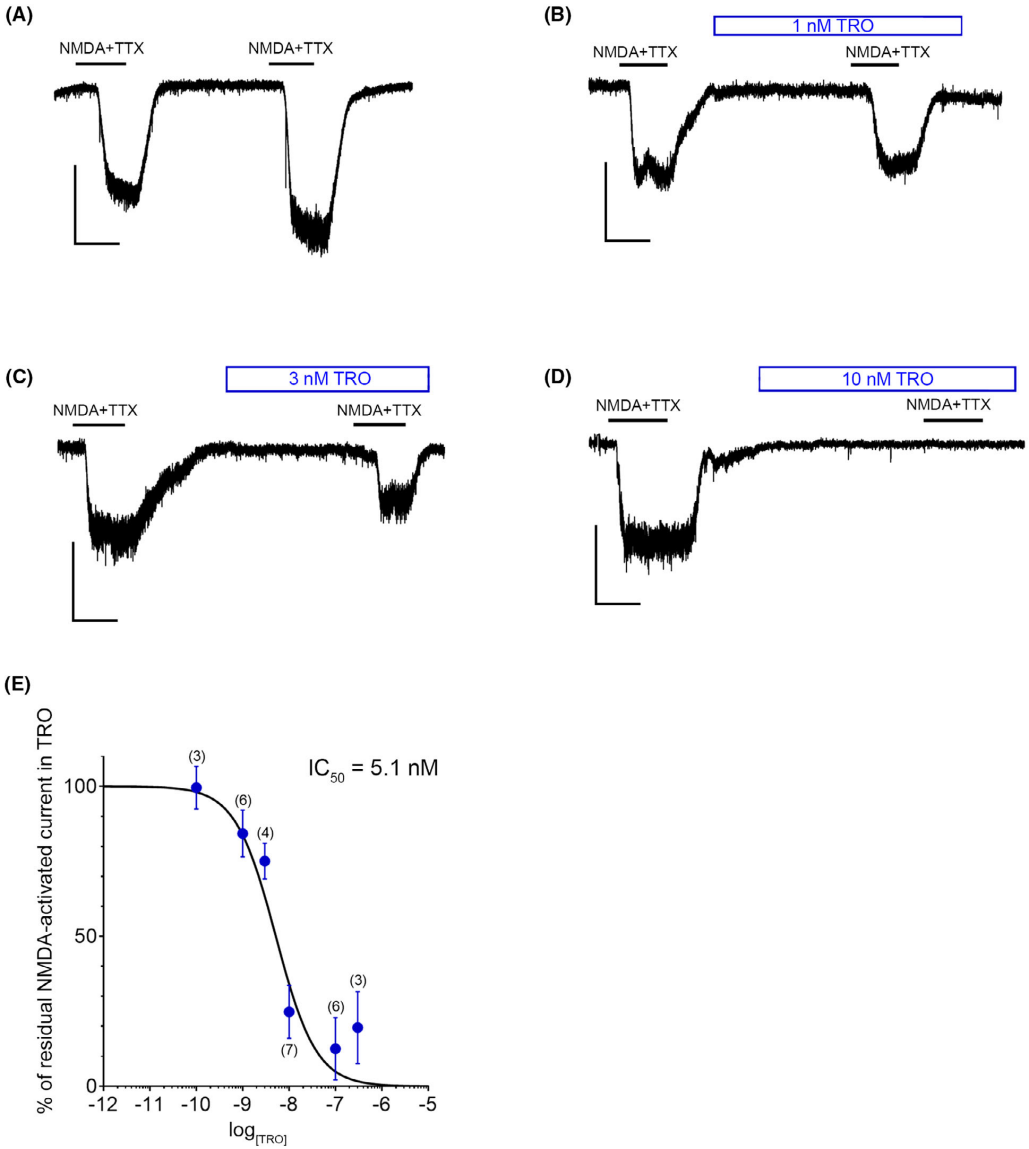
Patch clamp recordings of N‐methyl‐d‐aspartate receptor‐ (NMDAR‐) activated currents in primary rat hippocampal neurons in the absence or presence of different trodusquemine (TRO) concentrations. (A–D) Representative current traces recorded in cultured primary rat hippocampal neurons exposed twice to NMDA (100 μm, 2 min each, 7‐min interval). Tetrodotoxin (TTX; 200 nm) was co‐applied with NMDA to avoid excessive neuronal firing. Glycine (10 μm) was applied throughout the experiment. The second NMDA application was performed either in the absence (A; *n* = 16) or presence of TRO, applied also 5 min before the agonist (1, 3, or 10 nm from B to D). Downward deflections are spontaneous synaptic currents. Scale bars: 100 pA, 100 s. (E) Concentration–response curve of TRO‐mediated inhibition of NMDA‐induced current, obtained by normalizing the current recorded during the second NMDA application *vs* the first (taken as 100%). An IC_50_ of 5.1 nm (95% confidence limits: 3.0–8.6 nm) was determined. The number in brackets indicates the number of experiments (*n*) performed in TRO. Error bars: SEM.

The Na^+^ channel tetrodotoxin (TTX) (200 nm) was co‐applied with NMDA to prevent excessive neuronal firing. Two consecutive NMDA applications (2 min each, 7 min interval) were performed in the same cell and, as shown in Fig. 8A, comparable inward currents were recorded in control experiments. On average, the first and second NMDA‐activated currents were 3.69 ± 0.60 and 4.01 ± 0.62 pA/pF, respectively (*P* = 0.5458, *n* = 16). When the second NMDA application was done in the presence of TRO (0.1–300 nm), also pre‐applied 5 min before and 1 min after NMDA, a concentration‐dependent inhibition of the NMDA‐activated current was observed (Fig. 8B–D). A concentration‐response curve was obtained by plotting the residual NMDA‐activated current in the presence of TRO, normalized over the first NMDA application without TRO (taken as 100%), *versus* TRO concentration on a logarithmic scale (Fig. 8E). The data points were fitted to the Hill equation, and an *IC*_*50*_ of 5.1 nm (95% confidence limits: 3.0–8.6 nm) was calculated (Fig. 8E).

### ENT‐03 inhibits NMDAR activation induced by its agonist NMDA

**Fig. 9:**
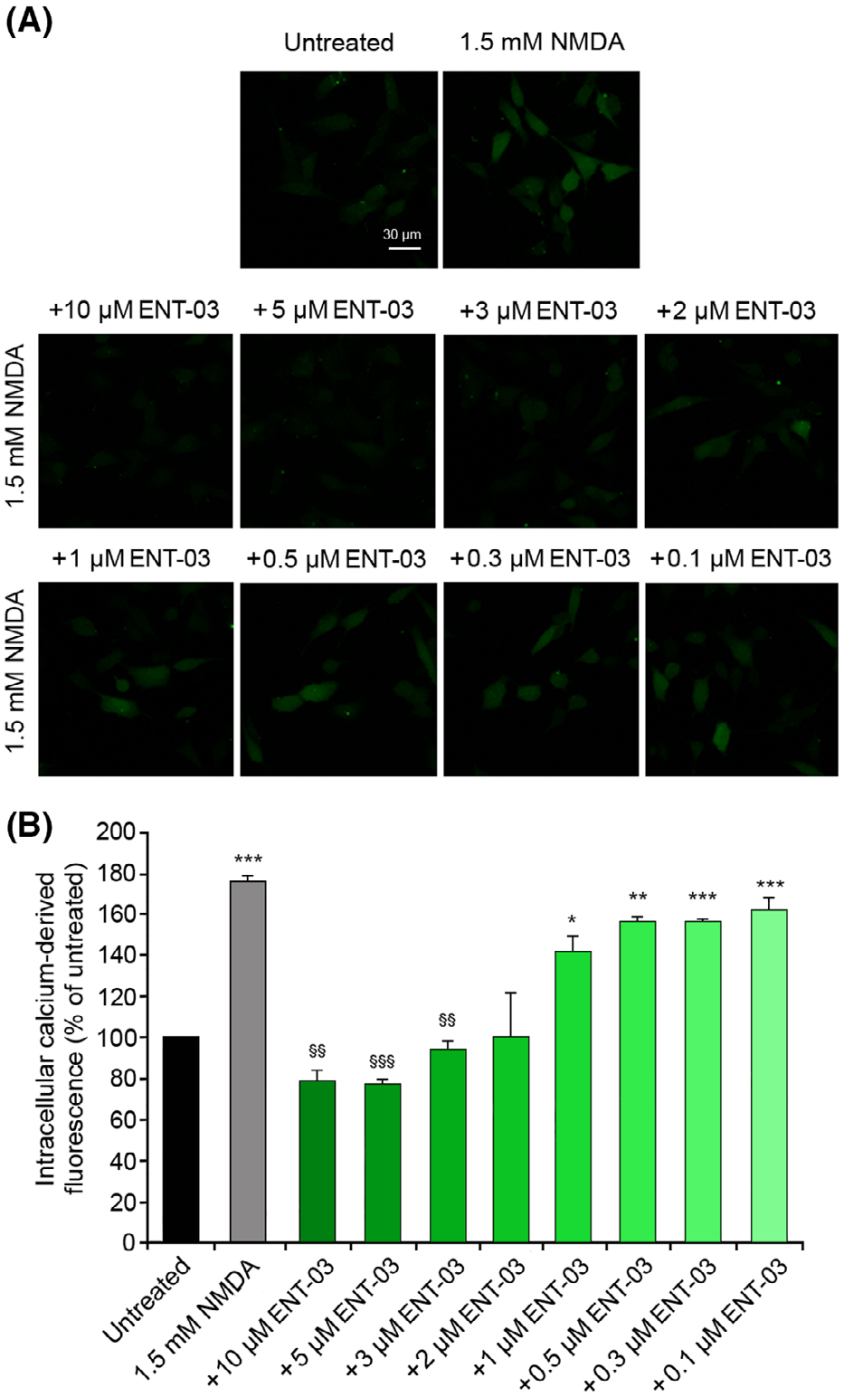
Intracellular free Ca^2+^ levels in SH‐SY5Y cells treated with N‐methyl‐d‐aspartate (NMDA) after treatment with different concentrations of ENT‐03. (A) Representative confocal scanning microscopy images of intracellular Ca^2+^ levels in untreated cells and cells treated with 1.5 mm NMDA for 10 min without or with pretreatment with 10, 5, 3, 2, 1, 0.5, 0.3, and 0.1 μm ENT‐03 for 10 min. Scale bar: 30 μm. (B) Semi‐quantitative analysis of intracellular free Ca^2+^‐derived fluorescence as shown in panel A. Variable numbers of cells (12–22) in three different experiments (*n* = 3) were analyzed for each condition, error bars are SEM. Comparisons between the different groups were performed by Student's *t*‐test, single (*), double (**), and triple (***) asterisks refer to *P* values <0.05, <0.01, and <0.001, respectively, relative to untreated cells. Double (§§) and triple (§§§) symbols refer to *P* values <0.01 and < 0.001, respectively, relative to NMDA treatment.

ENT‐03 is another aminosterol recently isolated from the mouse *M. musculus* [16], which shares with TRO very similar chemical characteristics (Fig. 1B,C). To investigate whether the inhibitory effect of TRO on the Ca^2+^ influx induced by NMDA was maintained using other aminosterols, we repeated the measurements of the intracellular Ca^2+^ levels in SH‐SY5Y cells after pretreating them with different concentrations of ENT‐03 and then treating them with 1.5 mm NMDA (Fig. 9). As observed in previous experiments (Fig. 2A,B), the treatment with only NMDA caused a significant influx of Ca^2+^ in the cytosol (Fig. 9). The pretreatment with high concentrations of ENT‐03 (2–10 μm) determined a reduction of this influx to levels similar to the untreated cells (Fig. 9). With lower ENT‐03 concentrations, the levels of Ca^2+^ increased again, until they reached with 0.5 μm ENT‐03 the levels observed with only NMDA without ENT‐03 (Fig. 9). These results indicate that the effect observed with TRO could also be obtained with other aminosterols with similar chemical structures.

### TRO has an inhibitory effect on AMPA and kainate receptors

**Fig. 10:**
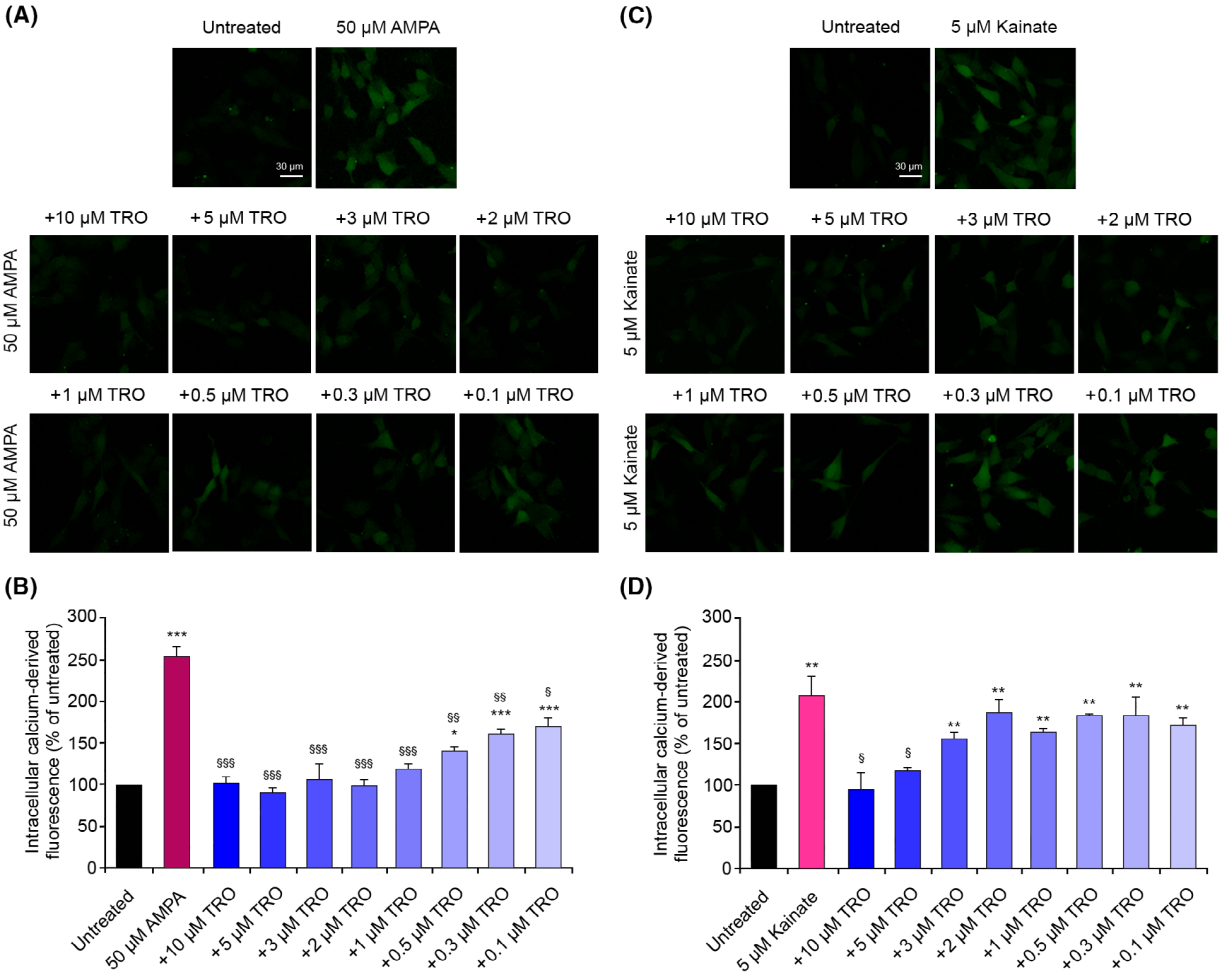
Intracellular free Ca^2+^ levels in SH‐SY5Y cells treated with AMPA and kainate after treatment with different concentrations of trodusquemine (TRO). (A, C) Representative confocal scanning microscopy images of intracellular Ca^2+^ levels in untreated cells and cells treated with (A) 50 μm AMPA or (B) 5 μm kainate for 10 min without or with pretreatment with 10, 5, 3, 2, 1, 0.5, 0.3, and 0.1 μm TRO for 10 min. Scale bar: 30 μm. (B, D) Semi‐quantitative analysis of intracellular free Ca^2+^‐derived fluorescence, as shown in panels A and C, respectively. Variable numbers of cells (12–22) in three different experiments (*n* = 3) were analyzed for each condition, error bars are SEM. Comparisons between the different groups were performed by Student's *t*‐test, single (*), double (**), and triple (***) asterisks refer to *P* values <0.05, <0.01, and <0.001, respectively, relative to untreated cells. Single (§), double (§§), and triple (§§§) symbols refer to *P* values <0.05, <0.01, and <0.001, respectively, relative to AMPA and kainate treatment.

Studies performed on PAS showed the ability of this compound to also inhibit the α‐amino‐3‐hydroxy‐5‐methyl‐4‐isoxazolepropionic acid receptors (AMPARs) and kainate receptors (KARs) besides NMDARs [11, 19]. Following the structural resemblance between PAS and aminosterols, we postulated that aminosterols also retain this ability. To investigate this possibility, we repeated the analysis by pretreating the SH‐SY5Y cells with different concentrations of TRO and then treating them with 50 μm AMPA or 5 μm kainate (Fig. 10). The treatment with only AMPA (Fig. 10A,B) and only kainate (Fig. 10C,D) showed a significant influx of Ca^2+^ in the intracellular space. The pretreatment with TRO before AMPA treatment had a marked inhibitory effect, even at the lowest TRO concentrations studied here (Fig. 10A,B). The pretreatment with TRO before kainate treatment also had a significant inhibitory effect, although at higher concentrations relative to AMPA (Fig. 10A,B). In the case of AMPA, the levels of Ca^2+^ remain similar to the untreated cells until 1 μm TRO, and with lower concentrations the influx increases, but never reached the levels of the only AMPA treatment without TRO (Fig. 10A,B). In the case of kainate, the Ca^2+^ ions significantly reduced their levels only with 10 and 5 μm TRO, increasing again at lower TRO concentrations. These results indicate that the inhibitory effect of TRO observed with NMDARs could also be applied to the other two ionotropic glutamate receptors, similarly to PAS.

## Discussion

### Aminosterols inhibit NMDARs with low nanomolar potency through direct binding

In this work, we have shown that TRO, used here as a representative natural aminosterol among those that have been more extensively studied, inhibits the NMDA‐induced and NMDAR‐mediated increase of intracellular Ca^2+^ in rat primary cortical neurons and human neuroblastoma SH‐SY5Y cells. The inhibition is dose‐dependent and is also confirmed, with a similar dose‐dependence, with the recently discovered natural mammalian ENT‐03. We investigated four hypotheses to explain the inhibition: (i) TRO‐mediated endocytosis of NMDARs, (ii) TRO‐mediated PTP1B inhibition through direct binding resulting in NMDAR inactivation, (iii) TRO‐membrane binding with cell membrane compression, and (iv) TRO‐NMDAR direct binding. The first three hypotheses are indirect mechanisms of NMDAR inactivation that have a rationale in well‐documented mechanisms previously described [25, 26, 27, 28, 29, 30, 40, 41, 42], but the results rule out all of them as mechanisms of TRO‐mediated NMDAR inhibition. By contrast, a FRET approach using a donor and acceptor located on NMDAR and TRO, respectively, shows a spatial proximal vicinity between the membrane protein tetramer and the small molecule, indicating direct binding between TRO and NMDARs. This finding is supported by the close chemical similarity between TRO and PAS, which is an established NMDAR inhibitor acting through direct binding after PAS adsorption to the cell membrane [11, 19].

Patch clamp experiments on primary rat hippocampal neurons indicate inhibition of NMDA‐induced currents, with an *IC*_*50*_ value of 5.1 nm (95% confidence limits 3.0–8.6 nm). The TRO‐mediated inhibition of NMDAR‐mediated increase of intracellular Ca^2+^ concentration, observed on neuroblastoma cells with confocal fluorescence microscopy and Fluo‐4 as an internalized Ca^2+^‐binding fluorescent probe, occurs at much higher concentrations, in the low micromolar range, but this is a common observation and arises from four main factors: the small amount of NMDARs in these cells relative to native neurons [30], the presence of pumps on the cell membrane and endoplasmic reticulum membrane that pump Ca^2+^ outside of the cytosol [43, 44, 45], the small entity of ryanodine‐sensitive Ca^2+^‐induced Ca^2+^ release in these nonexcitable cells [46], and differences in NMDAR subunit composition in SH‐SY5Y cells *versus* primary rat hippocampal neurons [47]. Electrophysiological methods measure directly the NMDA‐induced and NMDAR‐mediated ion currents and represent the technique of election for estimating *IC*_*50*_ values of NMDAR inhibitors [12, 44, 48].

### Rationale for aminosterol‐mediated inhibition of NMDARs in the low nanomolar range

We hypothesized that natural aminosterols could act as NMDAR inhibitors upon observing that these molecules are chemically related to PAS, another natural and well‐studied NMDAR inhibitor (Fig. 1D) [11, 19]. Structure–activity relationship (SAR) studies carried out on PAS showed that the substitution of the sulfate ester at C‐3 with a mono‐positively charged group decreased the *IC*_*50*_ value by *ca*. fivefold, from an approximate value of 25 μm for PAS to values of about 5 μm for PAS‐27 and PAS‐35 (Fig. 1D–F) [20]. The elongation of the charged group with the introduction of a progressively higher number of methylene groups, while maintaining the charge, also potentiates inhibition [21, 22]. The replacement of the acetyl group at C‐17 with a long alkyl moiety decreased the *IC*_*50*_ value by *ca*. 250‐fold, from 25 μm to 0.09 μm when using an isobutyl group (Fig. 1D,G) [23]. Therefore, with both chemical derivatizations the original PAS potency was increased substantially. Aminosterols have these substituents naturally with a pluri‐positive alkyl polyamine tail fused to the sterol C‐3 (either a spermine or spermidine) and an alkyl moiety of the cholestane type fused to C‐17 in the D‐ring (Fig. 1A–C). In the central 4‐ring structure, the only difference is a hydroxyl group present at C‐7 in aminosterols, which is absent in PAS and its derivatives (Fig. 1). Using the electrophysiological method of patch clamp applied to primary rat hippocampal neurons, the *IC*_*50*_ value of TRO was found here to be as low as 5 nm (95% confidence limits 2.9–9.0 nm), which is about 5000‐fold lower than that of PAS [20] and about 20‐fold lower than that of its more potent derivative [23].

In order for a ligand to be applicable as a potential drug, the *IC*_*50*_ value has to lie in a practical range. Excessively potent inhibitors, particularly when a significant pool is absorbed onto the membrane, would complicate the dosage. The use of aminosterols and their chemical optimization require therefore attention, but such a low affinity in the low nanomolar range has also been found for other lipophilic compounds in cell cultures or brain slices, including the Na^+^ channel blocker tetrodotoxin [49], the Ca^2+^ channel blocker ziconotide [50], or the water insoluble GluN2B inhibitor Radiprodil [51]. Given the involvement of NMDARs in a high number of diseases, as described in the following subsection, such a high potential inhibition may appear useful and exploitable in some cases.

Inhibition was also found with the mammalian natural aminosterol ENT‐03, which has chemical groups at C‐3 and C‐17 with the same stereospecificity as TRO in the SAR scheme of PAS (Fig. 1C). Moreover, the finding that TRO also inhibits AMPARs and KARs, in addition to NMDARs, is also confirmatory of the same type of inhibition as PAS, as the latter molecule was also found to inhibit all three ionotropic glutamate receptors [11, 19].

It is worth mentioning that *IC*_*50*_ measurements carried out in neurons may present concerns related to the heterogeneous subunit composition of the channel. As previously demonstrated [52], NMDARs expressed at the synapse in cultured hippocampal neurons are a majority GluN1/2A/2B triheteromers (~70%), with lower levels of GluN1/2A (~10%) and GluN1/2B (~20%) diheteromers, and each assembly could present subtle variations in pharmacology, as reported before [53].

Can we locate the binding site of TRO within NMDARs and provide a rationale of its high potency? Inspection of the known NMDAR structure, mode of interaction of TRO with the cell membrane and on the chemical similarity between TRO and PAS derivatives provide important hints. It has been reported that PAS needs to bind to the cell membrane to access its allosteric binding site within the NMDAR tetramer [11, 48]. It has also been suggested that the binding site is located at the level of the region including the TMD‐LBD linkers on the extracellular side of the cell membrane, in particular at the level of the extracellular‐membrane interface in the extracellular channel vestibule [11, 48].

**Fig. 11:**
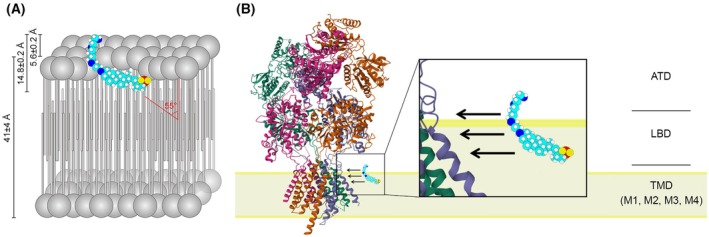
Schematic representation of the interaction of trodusquemine (TRO) with cell membranes and N‐methyl‐d‐aspartate receptor (NMDAR). (A) Representation of the insertion and localization of TRO within cell membranes resulting from experimental data and molecular dynamics simulations (adapted from [33]). Distances and angles indicated in the figure were measured experimentally [32]. (B) Schematic representation of the mode of approach of TRO to NMDAR (crystal structure of GluN1b‐GluN2B NMDA receptor structure in nonactive‐2 conformation 5FXI, adapted from [84]). The structure of NMDAR is represented embedded in the membrane, and its domains are indicated as extracellular amino‐terminal domain (ATD), extracellular ligand‐binding domain (LBD) and transmembrane domain (TMD), containing three transmembrane segments (M1, M3, and M4) and a re‐entrant pore loop (M2). The figure implies that TRO binds to the membrane and then approaches NMDAR for binding. It does not imply a precise molecular interaction.

TRO also interacts with cell membranes, and such interaction has been investigated in detail using TRO and liposomes in the form of large unilamellar vesicles (LUVs) as a model of the lipid bilayer of the cell membrane [18, 32, 33]. TRO stably binds to LUVs, as a molecule embedded within the hydrophilic portion of the first upper layer of the membrane, down to the interface between the hydrophilic and hydrophobic portions, exposing both sulfate and spermine groups to the aqueous phase (Fig. 11A) [32]. The molecule penetrates the lipid membrane by 9.2 ± 0.2 Å, with an angle of 55° with respect to the axis perpendicular to the bilayer plane, and it has its spermine moiety sticking out of the membrane by 5.6 ± 0.2 Å (Fig. 11A) [32]. TRO‐membrane binding was confirmed on cultured cells [32, 54].

Pre‐incubation of TRO with neurons is required to exhibit its inhibitory effect, most likely because a pre‐adsorption to the cell membrane and access to the NMDAR binding site via the membrane is necessary, as reported for PAS [11]. The location of TRO at the membrane with the spermine chain sticking out into the extracellular space suggests a binding site within the same TMD and TMD‐LBD linker region where a high density of negatively charged amino acid residues are located, making a highly favorable and cooperative electrostatic interaction with the positively charged TRO molecule (Fig. 11B). Indeed, in both human and rat GluN1 and GluN2A‐D, the three 15‐residue segments on the extracellular side before or after the M1, M3, and M4 segments that are annotated by the UniProt database as TMD helices, have together 7–9 negatively charged amino acid residues and only 1–3 positive residues, depending on subunit type. By contrast, TRO has 4 positively charged amino groups, all located in the spermine moiety and one negative sulfate on the other side of the molecule (Fig. 11B). The resulting highly favorable and cooperative electrostatic interaction between TRO and the channel vestibule of NMDAR can contribute to the high affinity of TRO for NMDARs and to its high potency (Fig. 11B), which is 20–16 000 times higher than that of any other TMD or TMD‐LBD binding inhibitors among those discovered so far [20, 23, 55, 56, 57, 58, 59] and within the same order of magnitude of the most potent inhibitors binding to the ATD or LBD domains [11, 12, 59, 60, 61, 62, 63].

Spermine, which is an important chemical portion of TRO, has been shown to be an activator of NMDARs in the high micromolar range by binding to the ATD of GluN1 subunits [62]. We can exclude that TRO has this effect on NMDARs because potent inhibition, rather than bland potentiation, has been found. More importantly, TRO has a relatively high affinity (low micromolar range) for the lipid bilayer of the cell membrane and, following a comparison with PAS, also for TMD and TMD‐LBD linkers of the NMDARs in the low nanomolar range, making it difficult for the spermine moiety of TRO to reach the ATD of NMDARs that is spatially distant and has a much lower binding affinity [62].

### Determinants of the pharmacology of TRO and its therapeutic potential

Following either systemic or intracerebral administration, TRO localizes principally to the hypothalamus, including regions involved in food intake and energy homeostasis [64], and ENT‐03 was recently shown to localize to the arcuate nucleus of the hypothalamus, the choroid plexus, and cerebrospinal fluid [65]. TRO has been shown to inhibit hypothalamic PTP1B in obese rodents, thereby increasing the sensitivity of both the insulin and leptin receptors, correcting diabetes and obesity [41, 42]. The inhibition of NMDARs shown here suggests a second mechanism by which TRO administration induces a reduction in food intake and weight loss. Pharmacological inhibition of NMDARs in neurons within the hypothalamus causes reduced appetite and weight loss due in part to inhibition of the expression of the orexigenic peptide AGRP [66, 67]. Deletion of PTP1B within neurons expressing NMDARs and AGRP increases their responsiveness to insulin, markedly suppresses the expression of AGRP, and consequently, food intake [68]. Our previously reported observation that TRO treatment of obese, diabetic ob/ob mice was accompanied by a significant reduction in the expression of hypothalamic AGRP [64] can now be plausibly explained as a consequence of inhibition of either or both PTP1B and NMDARs.

TRO has also been shown to prevent cognitive deterioration and neuronal loss in the hAPP‐J20 amyloidogenic mouse model of AD. Treatment beginning at 4.5 months (1 month, 2.5 mg·kg^−1^ every 5 days) resulted in significant recovery of memory and learning behavior [69]. Inflammation and neuronal loss were reduced in the hippocampus [69]. Because the genetic deletion of neuronal PTP1B in this model provided cognitive and neuron‐sparing benefits similar to those observed with TRO, the positive effects could be ascribed to pharmacological PTP1B inhibition by TRO. However, given the efficacy of NMDAR antagonists in treating symptomatically AD [70], the NMDA inhibitory activity of TRO might also explain the benefits observed in the animal model.

In other studies, SQ and TRO were found to strongly alter amyloid fibril formation by α‐synuclein (αS) and the amyloid β (Aβ) peptide [18, 71, 72, 73, 74] and inhibit the interaction of misfolded protein oligomers, including oligomers of Aβ and αS and the model protein HypF‐N, with biological membranes of both cultured cells and reconstituted liposomes [18, 71, 72, 73, 74, 75, 76]. Therefore, in addition to the effects of TRO that result from the inhibition of PTP1B and/or NMDARs, the interaction of this aminosterol with the lipid bilayer of cell membranes, with resulting inhibition of lipid‐induced protein aggregation and interaction of misfolded protein oligomers with the cell membranes, can contribute to its observed pharmacological properties [18]. At physiological pH, aminosterols exist as zwitterions with a net cationic charge. As a consequence, the aminosterol binds electrostatically to cell membranes containing negatively charged phospholipid and glycolipid head groups. The insertion of aminosterols into lipid bilayers leads to a decrease in its total negative charge, an increase in the mechanical resistance to indentation perpendicularly to the bilayer plane, and a reorganization of the spatial distribution of cholesterol (CHOL) and the monosialotetrahexosylganglioside 1 (GM1), all contributing to the reduced affinity of the misfolded oligomers for the membrane [32, 33].

The results presented in this work reveal another novel effect of aminosterols against neurodegeneration: the ability to inhibit NMDARs at nanomolar concentrations, as well as AMPARs and KARs. NMDARs are activated by membrane‐bound misfolded oligomers of Aβ_42_ [24, 30, 77, 78, 79, 80] via a mechanosensitive mechanism [30, 31]. In the context of neurodegeneration, it would have been interesting, for this specific study, to test the aminosterol‐mediated inhibition of NMDARs activated by membrane‐bound Aβ_42_ oligomers, but the binding itself of Aβ_42_ oligomers to the cell membrane is known to be inhibited by membrane‐bound aminosterols [71, 72, 76]. As a consequence of these competing effects, any aminosterol‐mediated inhibition of Aβ_42_‐induced activation of NMDARs would result primarily from a cell membrane displacement effect of membrane‐bound Aβ_42_ oligomers by aminosterols. For this reason, we have not tested Aβ_42_ oligomers for NMDAR activation, but rather used NMDA as an NMDAR activator.

## Conclusions

We have revealed that natural aminosterols can act as potent inhibitors of NMDARs, as well as possibly other ionotropic glutamatergic receptors such as AMPARs and KARs, with a potency in the low nanomolar range. Similarly to PAS, they act by binding to the cell membranes and then reaching their binding site on NMDARs, likely located at the level of the TMD and TMD‐LBD linker regions. The potency of TRO (electrophysiologically determined *IC*_*50*_) is 5000‐fold higher than that of PAS [20] and 20 to 16,000 times higher than those of the plethora of TMD or TMD‐LBD linker binding inhibitors reported so far [20, 23, 55, 56, 57, 58, 59]. The precise mechanism through which TRO and other aminosterols bind to NMDARs, the precise location of their binding site, and the NMDAR subunit selectivity remains unknown and will be the subject of future investigation, but the low nanomolar range of these compounds, and possibly other related derivatives, makes this class of molecules highly promising for the cure of one or more diseases associated with aberrant NMDAR function.

## Materials and methods

### Cell cultures

Gibco authenticated Primary Rat Cortex Neurons (A1084001; Life Technologies/Thermo Fisher Scientific, Waltham, MA, USA) were maintained in neuronal basal plus medium (Life Technologies/Gibco, Thermo Fisher Scientific, Waltham, MA, USA) supplemented with 0.5 mm GlutaMAX (Gibco) and 2% (v/v) B‐27 serum‐free complement (Gibco). Cell cultures were maintained at 37 °C in a 5.0% CO_2_ humidified atmosphere. Every 3 days, the medium was partially replaced with a fresh one. All experiments were performed 12–16 days after plating. Authenticated human SH‐SY5Y neuroblastoma cells (RRID:CVCL_0019) were purchased from A.T.C.C. (CRL‐2266, Manassas, VA, USA) and cultured in Dulbecco's modified Eagle's medium (DMEM), F‐12 HAM with 25 mm N‐2‐hydroxyethylpiperazine‐N‐2‐ethanesulfonic acid (HEPES) and NaHCO_3_ (1:1), supplemented with 10% fetal bovine serum (FBS), 2 mm glutamine, and 1% antibiotics, and routinely tested to ensure that they were free from mycoplasma contamination. Cell cultures were maintained at 37 °C in a 5% CO_2_ humidified atmosphere and grown until they reached 80% confluence for a maximum of 25 passages.

### Measurement of cytosolic free Ca^2+^ levels

Cytosolic Ca^2+^ levels were measured in living primary rat cortical neurons and SH‐SY5Y cells using 5 μm Fluo‐4 AM (Life Technologies/Thermo Fisher Scientific) added over the last 10 min of each treatment. The detection was performed using a TCS SP8 scanning confocal microscopy system equipped with an argon laser source (Leica Microsystems, Wetzlar, Germany) after excitation at 488 nm. A series of 1 μm thick optical sections (1024 × 1024) was taken through the cell depth for each sample using a Leica Plan Apo 63× oil immersion objective and projected as a single composite image by superimposition (Leica Microsystems). Three different experiments of each treatment were performed and normalized using the untreated cells. All the analyses were carried out using imagej software.

In one set of experiments, primary rat cortical neurons, plated in a 12‐well plate containing coverslips coated with poly‐d‐lysine at the density of 150 000 cells per well, were treated with 1.5 mm NMDA for 10 min without or with a 10 min pretreatment with 15, 10, 7.5, and 5 μm TRO. In a second set of experiments, SH‐SY5Y cells were plated in a 12‐well plate containing coverslips at the density of 120 000 cells per well and were treated with 1.5 mm NMDA for 10 min without or with a 10 min pretreatment with 10, 5, 3, 2, 1, 0.5, 0.3, and 0.1 μm TRO. Before the treatment with 1.5 mm NMDA for 10 min, the cells were also pretreated with 5 μm InSolution PTP1B inhibitor (539749; Merck, Darmstadt, Germany) for 2 h, with 10 μm memantine (Mem) for 1 h, and with 10, 5, 3, 2, 1, 0.5, 0.3, and 0.1 μm ENT‐03 for 10 min. In other sets of experiments, SH‐SY5Y cells were treated with 50 μm AMPA or 5 μm kainate for 10 min (rather than NMDA) without or with the pretreatment of 10 min with 10, 5, 3, 2, 1, 0.5, 0.3, and 0.1 μm TRO.

### Measurement of intracellular reactive oxygen species (ROS) levels

Intracellular ROS levels were measured in SH‐SY5Y cells using 5 μm chloromethyl derivative of 2′,7′‐dichlorodihydrofluorescein diacetate (CM‐H2DCFDA) (Life Technologies/Thermo Fisher Scientific) added over the last 15 min of each treatment. The detection was performed after excitation at 488 nm by a TCS SP8 scanning confocal microscopy system equipped with an argon laser source (Leica Microsystems) as described for the intracellular Ca^2+^ measurements. Three different experiments of each treatment were performed and normalized using the untreated cells. All the analyses were carried out using imagej software. SH‐SY5Y cells were seeded in a 12‐well plate containing coverslips at a density of 120,000 cells per well and treated with 1.5 mm NMDA for 30 min without and with a 10 min pretreatment with 10, 5, 3, 2, 1, 0.5, 0.3, and 0.1 μm TRO.

### GluN2B expression and RNA interference

SH‐SY5Y cells were plated in 12‐well plates containing coverslips at a density of 120 000 cells per well. Cells were fixed with methanol at −20 °C for 15 min, then washed three times with washing buffer (PBS and 0.1% Triton‐X) with agitation for 5 min each and incubated for 1 h with blocking buffer (washing buffer and 5% FBS). Afterwards, the cells were stained overnight at 4 °C with a 1:300 diluted mouse monoclonal anti‐GluN2B antibody labeled with Alexa Fluor‐488 (Santa Cruz Biotechnology, Dallas, TX, USA) in PBS and 1% FBS. The next day, the cells were washed with washing buffer and then detached. The levels of GluN2B were evaluated by the same TCS SP8 system described above (Leica Microsystems), after excitation at 488 nm. A series of 1 μm thick optical sections (1024 × 1024) was taken through the cell depth for each sample using a Leica Plan Apo 63× oil immersion objective and projected as a single composite image by superimposition (Leica Microsystems). Three different experiments of each treatment were performed and analyzed using imagej software.

In one set of experiments, GluN2B expression was evaluated with and without a pretreatment of 10 min with 5 μm TRO. In another set of experiments, cells were washed with PBS and transfected using 25 nm Stealth RNAi™ siRNA against GluN2B (Life Technologies/Thermo Fisher Scientific), 7 μL of lipofectamine, and 10 μL of 5 mg·L^−1^ transferrin in DMEM for 3 h in a 5% CO_2_ humidified atmosphere at 37 °C. The cells were also transfected with vehicle (transfection mix without siRNA) and with 25 nm Stealth RNAi™ negative controls siRNA (Life Technologies/Thermo Fisher Scientific). 3 h after transfection, DMEM was replaced with fresh complete medium, and after the 72 h incubation, the GluN2B levels were evaluated.

### TMA‐DPH anisotropy

SH‐SY5Y cells, seeded on 12‐well plates at a density of 120 000 cells per well, were loaded with 2 μm TMA‐DPH, with or without a treatment with 2 μm lysophosphatidylcholine (LPC) for 2 h, 10 μm arachidonic acid (AA) for 2 h, 0.5 mm cholesterol (CHOL) for 3 h, or 5 μm TRO for 10 min. The different incubation times derive from specific protocols for their incorporation into the phospholipid bilayer [30, 54]. Cells were then recovered after trypsination using PBS with 0.1 g·L^−1^ MgCl_2_ and 0.133 g·L^−1^ CaCl_2_ and put in a 10 × 4 path length quartz cuvette. The fluorescence anisotropy (*r*) values were then measured at 430 nm, exciting the samples at 355 nm, in an Agilent Cary Eclipse spectrofluorimeter (Agilent Technologies, Santa Clara, CA, USA) equipped with a thermostated cell holder attached to an Agilent PCB 1500 water Peltier system.

### Labelling of TRO with Alexa Fluor 594

TRO was synthesized as reported in previous works and stored as a powder at −20 °C [81]. The aminosterol was dissolved in distilled water to prepare a 100 mm stock solution, which was then stored at 4 °C. The fluorescent dye Alexa Fluor® 594 NHS Ester (Thermo Fisher Scientific) was solubilized in DMSO to prepare a 10 mm stock solution and stored at −20 °C. For the labeling process, 5 mm TRO and 0.5 mm fluorescent dye were incubated at 25 °C for 2 h in 0.1 m sodium bicarbonate buffer, pH 7.0, in a final volume of 20 μL under mild orbital shaking, as previously reported [32, 33]. The obtained solution had a ratio of labeled: total TRO of 1:10 and was directly used after incubation. As previously ensured with mass spectroscopy [32], no unreacted dye remains in solution after the labeling procedure.

### Fluorescence resonance energy transfer (FRET)

SH‐SY5Y cells, seeded on 12‐well plates at the density of 120 000 cells per well, were treated with Alexa Fluor‐594‐labeled TRO for 10 min, as acceptor (A), washed with PBS, and then fixed with methanol at −20 °C for 15 min. After three washes with washing buffer (PBS and 0.1% Triton‐X) with agitation for 5 min each, an incubation with blocking buffer (washing buffer and 5% FBS) for 1 h was performed. The cells were then stained overnight at 4 °C with a 1:300 diluted mouse monoclonal anti‐GluN2B antibody labeled with Alexa Fluor‐488 (Santa Cruz Biotechnology) in PBS and 1% FBS, as donor (D). The next day, cells were washed three times with washing buffer and then detached, as described. A negative control was also performed using the same protocol described above, with the only difference of treating the cells with 1 μg·mL^−1^ lipopolysaccharides (LPS) labeled with Alexa Fluor‐594 (Life Technologies/Thermo Fisher Scientific), 7 μL of lipofectamine, and 10 μL of 5 mg·L^−1^ transferrin in DMEM for 1 h, as acceptor (A).

Cells were analyzed using the Leica TCS SP8 system described above and previously [82]. The optical sections were acquired in the donor channel (excitation 488 nm, emission 499–535 nm), in the acceptor channel (excitation 561 nm, emission 640–700 nm) and in the FRET channel (excitation 488 nm, emission 640–700 nm). FRET efficiency (*E*) was calculated as:

(1) $$E=1-\frac{F_{\mathrm{DA}}}{F_{D}}$$

where *F*_*DA*_ is the fluorescence intensity of *D* in the presence of *A*, and *F*_*D*_ is the fluorescence intensity of *D* when *A* is far away.

### Electrophysiology

Wistar rats (Envigo, Italy) pups (postnatal days 0–1: P0–1) of both sexes were used for the preparation of primary cultures of hippocampal neurons, as described [83]. All animal experiments satisfied the regulatory requirements of the European Parliament (Directive 2010/63/EU), European Union Council (September 22, 2010), and Italian Animal Welfare Law (DL 26/2014). The protocol received approval from the Institutional Animal Care and Use Committee (Univ. Florence) and the Italian Ministry of Health. Animals were housed in the Ce.S.A.L. (Centro Stabulazione Animali da Laboratorio, University of Florence) in standard cages and maintained at 23 ± 1 °C with a 12‐h light/dark cycle, light on at 7 a.m., fed by a standard laboratory diet and tap water *ad libitum*. Animal suffering and the animal number required for data reproducibility were minimized, accordingly with the Ministerial notification (ser. no. 211/2022).

Briefly, hippocampi were isolated and cultured (1 × 10^4^ cells·well^−1^) in Neurobasal™ medium supplemented with B‐27 (Gibco), 100 ng·mL^−1^ nerve growth factor (NGF; Thermo Fisher Scientific), 100 U·mL^−1^ penicillin, 100 U·mL^−1^ streptomycin, and 2 mm l‐glutamine on glass coverslips coated with poly‐l‐lysine (8.3 mm) and laminin (5 mm) for at least 6 days *in vitro* (DIV6) before performing patch clamp recordings. At DIV3, 2.5 mm cytosine β‐d‐arabinofuranoside hydrochloride (ARA‐C: Merck) was added to the growth medium and removed after 24 h to inhibit glial cell proliferation. Whole‐cell patch clamp recordings were performed on primary cultures of rat hippocampal neurons as described previously [83]. The extracellular solution was 147 mm NaCl, 4 mm KCl, 1 mm MgCl_2_, 2 mm CaCl_2_, 10 mm D‐glucose, 10 mm 4‐(2‐hydroxyethyl)‐1‐piperazineethanesulfonic acid (HEPES), pH 7.4 (with NaOH). The intracellular solution was 130 mm K‐gluconate, 4.8 mm NaCl, 2 mm MgCl_2_, 1 mm CaCl_2_, 2 mm Na_2_‐ATP, 0.3 mm Na_2_‐GTP, 3 mm EGTA, 10 mm HEPES, pH 7.4 (with NaOH). The extracellular solution was supplemented with the NMDAR co‐agonist glycine (10 μm). NMDA (100 μm) was co‐applied with the most used selective Na^+^ channel blocker, TTX (200 nm) to avoid excessive firing and neurotransmitter release from nearby neurons. In our experimental conditions, the calculated liquid junction potential was −14 mV, a value that was taken into account in voltage measurements.

Cells were transferred in a 1‐mL platform‐mounted recording chamber in an inverted microscope (Olympus CKX41; Leica Microsystem) and superfused at a flow rate of 1.5 ml·min^−1^ by a 6‐way perfusion valve controller (Harvard Apparatus, Cambridge, MA, USA). Borosilicate glass electrodes (Harvard Apparatus) were pulled with a Sutter Instruments puller (model P‐87) to a final tip resistance of 3–4.5 MΩ. Data were acquired by an Axopatch 200B amplifier (Axon Instruments, Union City, CA, USA), low‐pass filtered at 10 kHz, stored, and analyzed by pClamp 9.2 software (Axon Instruments). All the experiments were performed at room temperature (20–22 °C). Series resistance (Rs) and membrane capacitance (Cm) were routinely measured by fast hyperpolarizing voltage pulses (from −60 to −70 mV, 40‐ms duration) and compensated. Only cells showing Cm and Rs variations <20% throughout the experiment were included in the analysis. After seal breaking through, the amplifier was shifted into the current‐clamp mode to verify the presence of action potentials (APs) in the recorded neuron: a series of current steps (2 pA increment) was applied from −2 pA to the appearance of the first/s AP/s (1.2 s step duration). Only cells showing mature (i.e., at least 20 mV overshoot) APs were included in the analysis. NMDA‐mediated currents were measured in −60 mV‐clamped cells as inward currents elicited by 100 μm NMDA superfusion. Two consecutive NMDA applications (100 μm, 2 min each, 7‐min interval) were performed in each cell. When indicated, TRO was added to the extracellular solution 5 min before, during and 1 min after the second NMDA application at different concentrations (0.1–300 nm). NMDA‐mediated currents were quantified as the difference between the baseline current (last 20 s before NMDA application) and the current measured during the last 20 s of NMDA challenge, and expressed as current density (pA/pF) after normalization to respective cell capacitance in averaged results. The percent inhibition of the second *vs* the first NMDA‐activated current was quantified, either in the absence or presence of TRO and expressed as ‘% of residual NMDA‐activated current’ in pooled data. Experiments were performed on 45 cells, isolated from 6 animals, having a resting membrane potential of −65.96 ± 1.30 mV, a membrane capacitance of 26.84 ± 1.46 pF and a membrane resistance of 297.40 ± 30.85 MΩ.

### Statistical analysis

All data were expressed as means ± SEM (standard error of the mean). Numbers of experiments (*n*) were indicated in each figure legend. Comparisons between the different groups were performed by Student's *t*‐test. The single (*), double (**), and triple (***) asterisks refer to *P* values <0.05, <0.01, and <0.001, respectively.

## Conflict of interest

The authors declare the following competing interests: MZ and DB are inventors in patents for the use of aminosterols in the treatment of Alzheimer's and Parkinson's diseases and are co‐founders and stockholders in Enterin, Inc. The remaining authors declare no competing interests.

## Author contributions

GF: investigation, validation, formal analysis, visualization, writing—original draft, writing—review and editing. EC: methodology, investigation, validation, formal analysis, visualization, writing—original draft, writing—review and editing. SE: investigation, visualization, writing—review and editing. FChe: investigation. MG: investigation. DB: writing—review and editing, resources. MV: writing—review and editing, methodology. MZ: writing—review and editing, funding acquisition, resources. AMP: Investigation, methodology, writing—review and editing, funding acquisition. FChi: conceptualization, methodology, writing—original draft, writing—review and editing, supervision, project administration, funding acquisition.

## Peer review

The peer review history for this article is available at https://www.webofscience.com/api/gateway/wos/peer‐review/10.1111/febs.70072.

## Data Availability

The raw data that support the findings of this study are available from the corresponding author [fabrizio.chiti@unifi.it] upon reasonable request.
